# Acute Brachial Artery Thrombosis in a Neonate Caused by a Peripheral Venous Catheter

**DOI:** 10.1155/2014/368256

**Published:** 2014-06-30

**Authors:** Simon Berzel, Emilia Stegemann, Hans-Joerg Hertfelder, Katja Schneider, Nico Hepping

**Affiliations:** ^1^Department of Neonatology, GFO-Hospitals Bonn, St Mary's Hospital, Robert-Koch Street 1, 53115 Bonn, Germany; ^2^Department of Vascular Surgery, GFO-Hospitals Bonn, St Mary's Hospital, Bonn, Germany; ^3^Department of Cardiology, Pulmonology and Angiology, University Medical Centre, Dusseldorf, Germany; ^4^Department of Experimental Hematology and Transfusion Medicine, University Medical Centre, Bonn, Germany

## Abstract

This case describes the diagnostic testing and management of an acute thrombosis of the brachial artery in a female neonate. On day seven of life, clinical signs of acutely decreased peripheral perfusion indicated an occlusion of the brachial artery, which was confirmed by high-resolution Doppler ultrasound. Imaging also showed early stages of collateralization so that surgical treatment options could be avoided. Unfractionated heparin was used initially and then replaced by low-molecular-weight heparin while coagulation parameters were monitored closely. Within several days, brachial artery perfusion was completely restored. Acetylsalicylic acid was given for additional six weeks to minimize the risk of recurring thrombosis. If inadequately fixated in a high-risk location, a peripheral venous catheter can damage adjacent structures and thus ultimately cause arterial complications.

## 1. Introduction

Occlusion of venous and arterial vessels in childhood is a rare but serious complication. The incidence of neonatal thrombosis is 0.5 per 10,000 live births [1]. The in-hospital incidence of venous or arterial thrombosis is approximately 5.3 per 10,000 children, with increased risk in the neonatal and adolescent period [2].

As per definition, spontaneous thrombotic events are vascular occlusions without an underlying cause, whereas acquired thrombotic events require the presence of a predisposing disease or occur after a therapeutic or diagnostic vascular intervention.

Prothrombotic risk factors in neonates are congenital coagulation defects, maternal gestational diabetes, diabetic fetopathy, neonatal sepsis, necrotizing enterocolitis, asphyxia, polycythemia, and various metabolic diseases [3].

However, the majority of thrombotic events are caused by vascular catheters—more than 90% in neonates are associated with umbilical venous and/or arterial catheters as well as other central venous lines. An acute arterial thrombosis caused by a peripheral venous catheter is extremely rare and quite challenging to manage in a multidisciplinary approach.

## 2. Case

At 40 + 1/7 weeks of gestational age, a female neonate was born with a birth weight of 4,350 gm via spontaneous vaginal delivery. The pregnancy was uneventful except for a maternal insulin-dependent diabetes. The family history did not reveal any risk factors for hypercoagulability or thrombotic events. As the mother's vaginal smear was GBS-positive, she received a single dose of an antibiotic given intravenously during labor.

The newborn girl did well initially (APGAR 10/10/10; umbilical-artery pH 7,22) but developed hypoglycemia with a blood glucose level of 21 mg/dL after one hour of life, which prompted her transfer to the neonatal intensive care unit. She received glucose intravenously. As elevated inflammatory markers (IL-6 was 146 pg/mL) suggested a neonatal infection, antibiotic therapy (Ampicillin/Sulbactam and Tobramycin) was initiated.

On day two of life, the newborn's blood glucose levels normalized, so glucose infusion was stopped. She received antibiotics for a total of seven days. On day five of treatment, a new peripheral venous catheter (24G Abbocath) was placed at the right antecubital fossa. Approximately 48 hours later, her right hand and forearm showed acute signs of significantly decreased perfusion: pallor, capillary refill of greater than ten seconds, hypothermia, and no detectable radial or ulnar pulses. The PIV was immediately removed.

**Figure 1:**
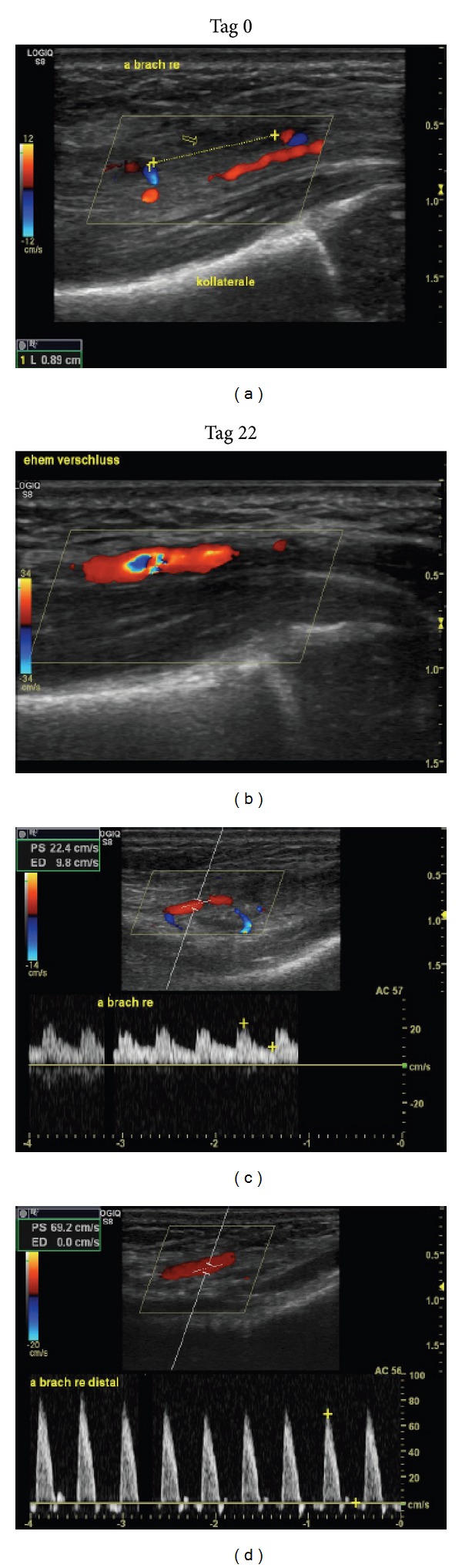
(a) Thrombosis of the right brachial artery at the time of diagnosis. (c) Corresponding severely diminished Doppler velocities distal to the site of occlusion (day 0). (b) Restored patency of the brachial artery (day 22). (d) Corresponding normalized Doppler pattern (day 22).

High-resolution Doppler ultrasound (GE S8, 18 MHz linear hockey stick transducer L8-18i) demonstrated complete occlusion of the distal brachial artery of 8.9 mm in length. Collateral vessels distal from the occlusion site showed a postocclusive, minimally pulsatile Doppler pattern (see Figure 1).

One hour after clinical signs had occurred, the fingertips of the right thumb and index finger developed a livid discoloration due to the predominating ulnar artery, and the remaining fingers continued to have a markedly prolonged capillary refill.

It was felt that the ultrasound images provided all the essential information needed for management decisions so that other imaging modalities such as phlebography or MRI angiography were not pursued, given that they carry significant inherent risks and potential negative side effects for neonates.

Early stages of collateralization and clinical improvement were reassuring, so neonatologists, angiologists, vascular surgeons, and hematology experts agreed on a conservative approach as there was no acute danger of limb loss.

Unfractionated heparin was intravenously given and adjusted to a maximum of 400 IU/kg/d while monitoring activated partial thromboplastin time (aPTT; 65 sec on second day of treatment; target aPTT 60–80 sec) and platelet count. Three days later, it was replaced by subcutaneous Enoxaparin (Clexane). An antifactor Xa level of 0.3 IU/mL was reached with a dose of 3 mg/kg/d on day five of treatment. Although the level was below the therapeutic target range, the dose was not increased further due to clinical improvement. The livid discoloration disappeared on day three of treatment; the capillary refill continued to improve and eventually normalized.

**Figure 2:**
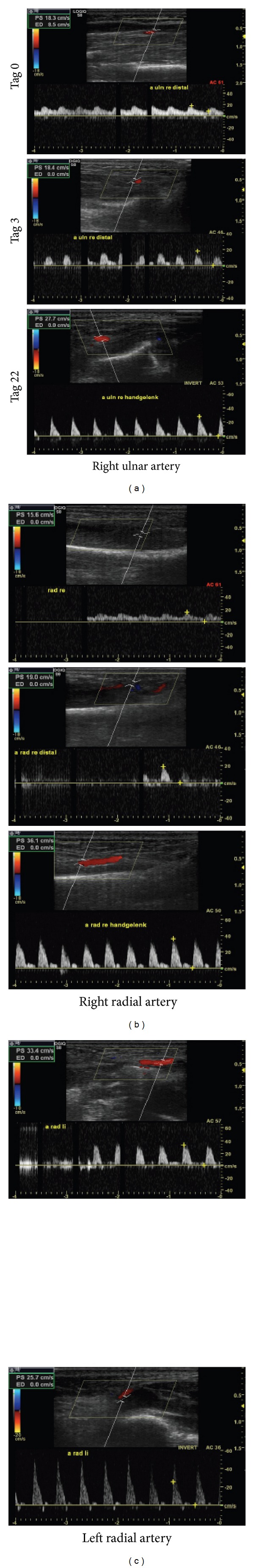
Velocities of the right radial and ulnar arteries (on day 0 and day 3 and after recanalization on day 22), compared to the left radial artery on days 0 and 22.

Daily follow-up ultrasound imaging showed progressing recanalization of the brachial artery (illustrated in Figure 2 on day seven after diagnosis). Clinically, radial and ulnar pulses were equally strong on both arms.

Platelet counts remained within the normal range for age and did not suggest heparin-induced thrombocytopenia (HIT). D-dimers showed an initial rise of up to 2.1 mg/L and quickly normalized under therapy. The girl was discharged home on day 18 of life. At that time, her right brachial artery was completely recanalized with unobstructed flow (see Figure 2).

Enoxaparin was given subcutaneously until day 20 of treatment; acetylsalicylic acid (ASA) was given in a dose of 3 mg/kg/d for six weeks to minimize the risk of recurring thrombosis. Extensive diagnostic testing (3 days after the thrombotic event) did not reveal any risk factors for hypercoagulability (e.g., deficiency of proteins C and S, antiphospholipid syndrome, and APC resistance).

The brachial artery remained patent beyond the end of therapy. Further clinical observation was done by the pediatrician in the routine preventive examinations.

## 3. Discussion

Children and adolescents tend to have thrombotic events much less often than adults. The highest risk occurs during the neonatal period, mainly in association with an umbilical venous or arterial catheter or other central venous lines [4]. Thromboembolism caused by a peripheral venous catheter is rare; however, placement can damage both the vein and adjacent tissues such as arteries or nerves.

The case above illustrates the serious complication of brachial artery thrombosis two days after placement of a peripheral venous catheter with subsequent acute ischemia of forearm and hand. It is thought that, because of the time interval between placement and clinical presentation, the peripheral venous catheter was properly placed initially but affected the adjacent artery with repetitive movements of the cubital joint. Unfortunately, at this time, the patient's joint was not stabilized as required. In order to minimize the risk of movement-related complications, arm boards are considered standard of care [5].

Early diagnosis, that is, recognizing the clinical presentation quickly and confirming the suspicion of acute thrombosis with imaging, is crucial for successful treatment and long-term prognosis. One can choose between ultrasounds with color Doppler, venous or arterial angiography, magnetic resonance angiography (MRA), or computed tomography angiography (CTA). With the exception of ultrasound, all other modalities are invasive and carry significant inherent risks and potential negative side effects, particularly for neonates.

Color Doppler ultrasound confirmed the diagnosis and provided all the information necessary for treatment, including clear visualization of collateral vessels. The chance of successful and complete recanalization is highest in the first few days of life in comparison to other age groups, making invasive treatment options for neonates extremely rare. Management should be aimed at reducing thrombus growth, with recanalization of the affected artery being the ultimate treatment goal.

The most recent practice guidelines for pediatric patients are based on adapted treatment regiments for adults. Unfractionated heparin (UFH) is highly recommended as first choice in acute situations [6].

Activated partial thromboplastin time (aPTT) is the most important parameter for monitoring, provided the antithrombin (AT) level is within normal range for age. Therapeutic intravascular lysis or surgical procedures are considered high risk and are therefore only indicated if there is acute danger of limb loss or potential hemodynamic compromise.

The use of UFH is usually followed by LMWH (monitored by antifactor Xa-levels), the subcutaneous application of which can be continued as outpatient therapy. Other advantages include reducing the risk for heparin-induced thrombocytopenia and not requiring any venous catheter for its application.

Moreover, any arterial thrombosis warrants an interdisciplinary discussion about the prophylactic use of antiplatelet medications such as ASA for the individual patient. The duration of ASA therapy for 6 weeks was a clinical decision made in this individual case in the absence of risk factors.

## 4. Conclusions

Ultrasound is an essential imaging modality used in neonatal intensive care medicine. Our case illustrates how high-resolution color Doppler ultrasound served as a noninvasive diagnostic tool in a seriously sick newborn and as a monitoring device for successful treatment progress.

Anticoagulation with low-molecular-weight heparin should be adjusted based on antifactor Xa-levels. D-dimers are used as an indicator of successful resolution of thrombotic material [7].
